# Developing a cancer‐specific trigger tool to identify treatment‐related adverse events using administrative data

**DOI:** 10.1002/cam4.2812

**Published:** 2020-01-03

**Authors:** Saul N. Weingart, Jason Nelson, Benjamin Koethe, Omar Yaghi, Stephan Dunning, Albert Feldman, David M. Kent, Allison Lipitz‐Snyderman

**Affiliations:** ^1^ Tufts Medical Center Boston MA USA; ^2^ Department of Medicine Tufts University School of Medicine Boston MA USA; ^3^ OptumLabs Cambridge MA USA; ^4^ Predictive Analytics and Comparative Effectiveness Center Tufts University School of Medicine Boston MA USA; ^5^ Memorial Sloan Kettering Cancer Center New York NY USA

**Keywords:** adverse event, epidemiology, oncology, patient safety, quality of care, trigger tool

## Abstract

**Background:**

As there are few validated tools to identify treatment‐related adverse events across cancer care settings, we sought to develop oncology‐specific “triggers” to flag potential adverse events among cancer patients using claims data.

**Methods:**

322 887 adult patients undergoing an initial course of cancer‐directed therapy for breast, colorectal, lung, or prostate cancer from 2008 to 2014 were drawn from a large commercial claims database. We defined 16 oncology‐specific triggers using diagnosis and procedure codes. To distinguish treatment‐related complications from comorbidities, we required a logical and temporal relationship between a treatment and the associated trigger. We tabulated the prevalence of triggers by cancer type and metastatic status during 1‐year of follow‐up, and examined cancer trigger risk factors.

**Results:**

Cancer‐specific trigger events affected 19% of patients over the initial treatment year. The trigger burden varied by disease and metastatic status, from 6% of patients with nonmetastatic prostate cancer to 41% and 50% of those with metastatic colorectal and lung cancers, respectively. The most prevalent triggers were abnormal serum bicarbonate, blood transfusion, non‐contrast chest CT scan following radiation therapy, and hypoxemia. Among patients with metastatic disease, 10% had one trigger event and 29% had two or more. Triggers were more common among older patients, women, non‐whites, patients with low family incomes, and those without a college education.

**Conclusions:**

Oncology‐specific triggers offer a promising method for identifying potential patient safety events among patients across cancer care settings.

## Graphical abstract

A claims‐based trigger tool was developed to identify potential adverse events and medical errors among 320 000 patients with breast, colorectal, lung, and prostate cancers. During an initial year of therapy, cancer‐specific trigger events affected 19% of patients and varied by disease and metastatic status, from 6% of patients with nonmetastatic prostate cancer to 41% and 50% of those with metastatic colorectal and lung cancers, respectively.

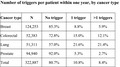

## 1 INTRODUCTION

Oncology care is an extraordinarily high‐risk activity, given the nature of the disease and its toxic therapies. While medical oncology was at the epicenter of the patient safety revolution with the 1994 overdose of Dana‐Farber Cancer Institute patient Betsy Lehman, there has been surprisingly little reliable and consistent information about patient safety in cancer care.^1^ Only three high‐quality studies of chemotherapy errors have been published to date, and virtually all patient safety‐related research performed in oncology settings has been conducted in regional or national referral centers.^2^, ^3^, ^4^ A literature review examining high‐quality studies of medication errors related to chemotherapy concluded that our ability to measure errors and injuries across the continuum of cancer care is poor at best.^5^

A variety of factors account for the dearth of robust research studies in cancer patient safety, including the physiologic vulnerability of cancer patients and the expected toxicities of many cancer‐directed therapies.^5^ Though successful in various medical settings^6^, ^7^, ^8^, ^9^, ^10^, ^11^ and in flagging potential diagnostic delays,^12^, ^13^ attempts to identify treatment‐related complications using so‐called “trigger tools” have worked poorly in cancer care.^14^ An oncology trigger tool piloted in the UK National Health Service showed poor performance characteristics, a rigorous French study examining a 22‐item trigger tool for adverse drug events showed low positive predictive values (PPVs),^15^, ^16^ and a Danish cancer center study showed disappointing interrater agreement, even with use of expert chart reviewers.^17^, ^18^

Without a robust measurement approach to patient safety in oncology that works across the continuum of oncology care, it is difficult to advise patients and their clinicians about the likely toxicities of therapy, the risk of treatment‐related errors, or the best site of care for their disease. Better measurement of adverse events (AEs) and medical errors could help medical and cancer center leaders to identify opportunities for improvement and inform programmatic priorities for policy makers. Most health‐care organizations use quality metrics appropriate for general medical patients to describe the quality of oncology care, but the applicability of commonly used metrics such as infection rates and readmissions apply poorly to oncology care. Efforts to assess cancer programs based on cancer registry data are limited to a small subset of analytic cases and outdated information. Creating a more streamlined and accessible approach to patient safety measurement for oncology would develop significant social value.

To address this problem, a team of researchers, oncology practitioners, and quality measurement and patient safety experts developed a set of oncology‐specific triggers using clinical data from patients at Memorial Sloan Kettering Cancer Center (MSK) undergoing an initial course of cancer‐directed therapy. Trigger tools use indicators, such as antidote medications, abnormal laboratory parameters, “stat” medication orders, and changes in the level of care, to signal the presence of a medical error or iatrogenic injury. Unlike previously published studies that failed to validate oncology‐specific trigger tools,^14^, ^15^, ^16^, ^17^, ^18^ the MSK team identified 49 high‐value oncology triggers with an overall PPV of 0.48 for AEs and 0.18 for preventable events using physician chart review as the gold standard.^4^, ^19^, ^20^

We undertook the present project in order to further develop the use of oncology‐specific triggers to identify treatment‐related AEs. Our project had three specific aims: (a) to construct a claims‐based trigger tool capturing the MSK triggers as International Classification of Diseases (ICD) and Current Procedural Terminology (CPT) codes, and (b) to examine the prevalence of trigger events among a commercial claims cohort. We hypothesized that it would be feasible to create a cancer‐specific claims‐based trigger tool, and that the prevalence of trigger events would vary by cancer type and metastatic status.

## 2 METHODS

### 2.1 Subjects

We selected a cohort of patients undergoing an initial course of cancer‐directed therapy for breast, lung, colorectal, and prostate cancers using the OptumLabs^®^ Data Warehouse (OLDW). OLDW includes de‐identified administrative claims and electronic health record (EHR) data on over 200 million patients, including claims for inpatient and ambulatory care for commercial and Medicare Advantage enrollees.^21^ It includes limited patient demographic information drawn from enrollment records. Socioeconomic status information in OLDW, including race/ethnicity, household income, and educational attainment, are imputed variables sourced from a national supplier of consumer marketing data. Mortality status is ascertained in OLDW through multiple sources including the Social Security Death Index, inpatient discharge status, and electronic medical records. We used ICD and CPT codes to select patients with cancer diagnoses who received cancer‐specific therapies including surgery, radiation therapy, or chemotherapy (infusion as well as oncolytic or hormonal therapies). Inclusion criteria included a new cancer diagnosis of breast, lung, colorectal, and prostate cancers from 1 January 2008 through 31 December 2014, with initiation of a cancer‐specific surgery, radiation, or chemotherapy during that period. To ensure a new cancer diagnosis, subjects with cancer diagnoses or treatments in 2005‐2007 and those with a cancer recurrence code were excluded.^22^, ^23^, ^24^

We abstracted sociodemographic characteristics (including age, gender, race/ethnicity, insurance (commercial, Medicare managed care), household income, and educational attainment), cancer diagnosis, and cancer‐specific therapies from the claims database, excluding cases of male breast cancers and subjects under age 18. We used a modified algorithm that excludes cancer as a comorbidity to calculate each patient's Charlson comorbidity index,^25^ an algorithm developed by Whyte and colleagues to classify cancer metastatic status,^26^ and the number of unplanned hospital admissions and inpatient days as an additional indicator of individuals at high‐risk of harm. We abstracted the dates associated with diagnosis and treatment codes, hospitalizations, and vital status.

### 2.2 Measurements

To define a set of oncology‐specific triggers, we identified ICD and CPT codes corresponding to 16 of the 23 highest (≥50%) PPV triggers from the MSK developmental study (Table 1). Triggers included events such as neutropenic fever, abnormal serum potassium or bicarbonate, return to the operating room or interventional suite within 30 days of surgery, initiation of therapeutic anticoagulation, and nephrology consultation. We recognized some inherent ambiguity in the use of ICD codes, as certain codes denote nonspecific laboratory abnormalities (eg, 790.6).

**Table 1:** Coding algorithm for selected oncology‐specific triggers

Trigger	Coding algorithm*
General care	
Pressure ulcer	ICD9 707.x (exclude 707.21 and 707.22)
Return to the operating room or interventional radiology within 30 d of surgery	See Osborne NH, et al
Vital signs	
Low oximetry results (SaO2 < 88%)	ICD9 799.02
Fever (> 38.2°C)	ICD9 780.6 and ICD9 288.x
Orders	
Blood transfusion	ICD9 V58.2, CPT 36 430
Contact precautions/order for isolation	ICD9 V07.x
Nasogastric tube (not in operating room)	ICD9 96.07, CPT 43 753
Non‐contrast chest CT after radiation to the chest	CPT 71 250
Percutaneous drain placement	ICD9 54.91, CPT 32 557
Laboratories	
Abnormal serum bicarbonate (< 18,> 36 mEq/L)	ICD9 790.6, 276.2, 276.3
Abnormal serum potassium (> 6, < 2.5 mEqL)	ICD9 276.8, 276.7
Clostridium difficile toxin positive	ICD9 008.45
Elevated creatinine > 1 mg/dL and 50% greater than baseline	ICD9 584.9 acute kidney injury (not present on admission)
Positive blood culture without contaminant	ICD9 790.7 bacteremia, CPT 87 040, 87 103
Medication‐related	
Initiation of therapeutic anticoagulation	medications: warfarin, enoxaparin, apixaban, rivaroxaban, dabigatran, fondaparinux, edoxaban
Consultations	
Nephrology consultation	E&M visit (outpatient CPT 99241‐99245, inpatient CPT 99251‐99255) and provider_specialty = ‘nephrologist’

Note

Osborne NH, Nicholas LH, Ryan AM, Thumma JR, Dimick JB. Association of hospital participation in a quality reporting program with surgical outcomes and expenditures for Medicare beneficiaries. *JAMA* 2015; 313:496‐504.

Abbreviations: CPT, Current Procedure Terminology; CT, computed tomography; ICD, International Classification of Diseases.

* ICD9 codes mapped to ICD10

To distinguish between complications related to a cancer‐specific treatment rather than the patient's cancer or non‐cancer comorbidities, we required a logical and temporal relationship between each trigger and its likely cause. We assumed that each trigger event would be temporally related to a specific exposure and that it would persist for a limited period of time. For example, neutropenic fever was associated with chemotherapy but not surgery or radiation. We assumed that neutropenic fever would follow within 30 days of chemotherapy and expect to persist for no more than 30 days. Recognizing the diversity of therapies and therapeutic regimens, we consulted with oncology clinicians to make generic assumptions about the most common and likely relationship of triggers and exposures, as shown in the Appendix.

### 2.3 Analyses

We characterized the cohort by sociodemographic and clinical characteristics and cancer‐specific treatments (surgery, radiation, and/or chemotherapy), stratified by cancer type (breast, colorectal, lung, prostate) and metastatic status.

We then tabulated the number and percent of patients with each AE trigger during a 1‐year period beginning with the date of the initial cancer‐directed therapy. We tabulated the number and percent of patients with no trigger events, one event, and two or more events. We performed separate analyses by cancer type and metastatic status. We examined the prevalence of trigger events by sociodemographic and clinical characteristics, using the Chi‐square and Wilcoxon rank‐sum test for categorical and continuous variables, respectively.

Analyses used SAS 9.4 for Windows (SAS Institute) and R 3.4.3 (The R Foundation). The study protocol was reviewed in advance by the Tufts Health Sciences Institutional Review Board (IRB) and determined to be exempt from human subjects review due to the use of a de‐identified dataset.

## 3 RESULTS

### 3.1 Cohort characteristics

The study cohort included 322 887 unique subjects with breast, colorectal, lung, and prostate cancers (Table 2). The mean age was 64, consistent with a commercially insured patient population. Males comprised a greater percentage of patients with colorectal and lung cancers than women. While the majority of patients were white, Asians, blacks, and Hispanics were also present. There was missing data regarding race/ethnicity, household income, and education for at least one‐third of the cohort.

**Table 2:** Cohort characteristics

Characteristic	Breast	Colorectal	Lung	Prostate	Overall
N	124 253	52 383	51 311	94 940	322 887
Age [mean (SD)]	59.5 (12.1)	63.2 (12.5)	67.1 (10.5)	66.9 (9.1)	63.5 (11.6)
Sex [n (%)]					
Male	–	27 616 (52.7%)	27 170 (53.0%)	94 940 (100.0%)	149 726 (46.4%)
Female	124 253 (100.0%)	24 767 (47.3%)	24 141 (47.0%)	–	173 161 (53.6%)
Race/Ethnicity [n (%)]					
Missing/Unknown	37 198 (29.9%)	18 961 (36.2%)	19 432 (37.9%)	32 741 (34.5%)	108 332 (33.6%)
Asian	2398 (1.9%)	862 (1.6%)	634 (1.2%)	1019 (1.1%)	4913 (1.5%)
Black	9543 (7.7%)	3847 (7.3%)	3538 (6.9%)	7920 (8.3%)	24 848 (7.7%)
Hispanic	5491 (4.4%)	2360(4.5%)	1271 (2.5%)	3463 (3.6%)	12 585 (3.9%)
White	69 623 (56.0%)	26 353 (50.3%)	26 436 (51.5%)	49 797 (52.5%)	172 209 (53.3%)
Annual household income [n (%)]					
Unknown	44 997 (36.2%)	22 584 (43.1%)	23 569 (45.9%)	37 349 (39.3%)	128 499 (39.8%)
<$25K	15 627 (12.6%)	6713 (12.8%)	8845 (17.2%)	10 733 (11.3%)	41 918 (13.0%)
$24K ‐ $149K	19 793 (15.9%)	8511 (16.2%)	8488 (16.5%)	15 694 (16.5%)	52 416 (16.2%)
$150K ‐ 249K	23 057 (18.6%)	8367 (16.0%)	6551 (12.8%)	17 314 (18.2%)	55 289 (17.1%)
$250K ‐ $499K	12 336 (9.9%)	3964 (7.6%)	2553 (5.0%)	8514 (9.0%)	27 367 (8.5%)
$500K+	8513 (6.9%)	2244 (4.3%)	1305 (2.5%)	5336 (5.6%)	17 398 (5.4%)
Education [n (%)]					
Missing/Unknown	34 322 (27.6%)	17 925 (34.2%)	18 449 (36.0%)	30 698 (32.3%)	101 394 (31.4%)
Less than 12th grade	217 (0.2%)	141 (0.3%)	89 (0.2%)	149 (0.2%)	596 (0.2%)
High school diploma	20 749 (16.7%)	9891(18.9%)	10 987 (21.4%)	16 468 (17.3%)	58 095 (18.0%)
Less than bachelor degree	48 901 (39.4%)	18 423 (35.2%)	17 365 (33.8%)	35 020 (36.9%)	119 709 (37.1%)
Bachelor degree plus	20 064 (16.1%)	6003 (11.5%)	4421 (8.6%)	12 605 (13.3%)	43 093 (13.3%)
Insurance type [n (%)]					
Private insurance	99 932 (80.4%)	42 456 (81.0%)	37 196 (72.5%)	69 486 (73.2%)	249 070 (77.1%)
Medicare Advantage	24 321 (19.6%)	9927 (19.0%)	14 115 (27.5%)	25 454 (26.8%)	73 817 (22.9%)
Clinical characteristics					
Metastatic disease [n (%)]	26 791 (21.6%)	18 671 (35.6%)	30 169 (58.8%)	10 800 (11.4%)	86 431 (26.8%)
Charlson index* [mean (SD)]	2.0 (1.6)	2.8 (1.9)	3.6 (1.8)	2.8 (1.4)	2.6 (1.7)
Rehospitalized w/in 1 y [n (%)]	36 780 (29.6%)	33 047 (63.1%)	32 092 (62.5%)	37 798 (39.8%)	139 717 (43.3%)
Hospital days [mean (SD)]	5.1 (8.8)	10.4 (13.9)	10.4 (12.9)	4.1 (7.9)	7.3 (11.4)
Treatment type [n (%)]					
Chemotherapy only	18 357 (14.8%)	8724 (16.7%)	11 501 (22.4%)	18 830 (19.8%)	57 412 (17.8%)
Radiation only	11 407 (9.2%)	1273 (2.4%)	7260 (14.1%)	21 253 (22.4%)	41 193 (12.8%)
Surgery only	22 952 (18.5%)	22 770 (43.5%)	6818 (13.3%)	31 598 (33.3%)	84 138 (26.1%)
Multimodality	71 537 (57.6%)	19 616 (37.4%)	25 732 (50.1%)	23 259 (24.5%)	140 144 (43.4%)

Note

Percentages may not add to 100% due to rounding.

* NIH measure for cancer patients’ modification. https://healthcaredelivery.cancer.gov/seermedicare/considerations/calculation.html.

Overall, 27% of patients had metastatic cancer. The percent of patients with metastatic disease varied from 22% for breast cancer to 59% for lung cancer. The cancer‐specific Charlson index suggested a moderate burden of comorbid non‐cancer illness. Forty‐three percent of patients received multimodality cancer therapy.

### 3.2 Trigger prevalence

Cancer‐specific trigger events were common, affecting 19% of patients over the initial 1‐year course of therapy (Table 3). The trigger burden varied by disease and metastatic status. Among patients with nonmetastatic disease, the prevalence of trigger events was greatest among patients with lung (33%) and colorectal (19%) cancers and least among those with prostate (6%) and breast (10%) cancers—likely a reflection of treatment types and toxicities as well as patients’ underlying physiologic reserve. There was a similar, but amplified, pattern among patients with metastatic disease. There was a particularly heavy burden of trigger events among those with lung (50%) and colorectal (41%) cancers, although patients with metastatic breast (31%) and prostate (25%) cancers also experienced significant treatment‐related morbidity. The most prevalent triggers were abnormal serum bicarbonate, blood transfusion, non‐contrast chest CT scan following radiation therapy, hypoxemia, contact precautions, neutropenic fever, and abnormal serum potassium.

**Table 3:** Trigger prevalence within 1 y, by cancer type and metastatic status

Trigger	Breast cancer n = 124 253		Colorectal cancer n = 52 383		Lung cancer n = 51 311		Prostate cancer n = 94 940	
	Nonmetastatic n = 97 462	Metastatic n = 26 791	Nonmetastatic n = 33 712	Metastatic n = 18 671	Nonmetastatic n = 21 142	Metastatic n = 30 169	Nonmetastatic n = 84 140	Metastatic n = 10 800
Any trigger	10.2% (9958/97 462)	30.9% (8275/26 791)	19.2% (6488/33 712)	41.4% (7738/18 671)	32.8% (6930/21 142)	50.2% (15 157/30 169)	5.9% (4987/84 140)	24.6% (2654/10 800)
Anticoagulation	0.1% (77/87 345)	0.2% (56/25501)	0.2% (63/32 893)	0.5% (97/18 217)	0.3% (49/17 799)	0.4% (109/26 252)	0.1% (49/63 869)	0.2% (17/9818)
Bacteremia/positive blood culture	0.3% (279/87 345)	1.4% (361/25 501)	1.1% (355/32 893)	3.1% (571/18 217)	1.6% (279/17 799)	2.4% (617/26 252)	0.3% (183/63 869)	1.6% (154/9818)
Abnormal serum bicarbonate	1.8% (1575/87 345)	5.0% (1285/25 501)	3.9% (1287/32 893)	9.3% (1700/18 217)	4.7% (831/17 799)	7.3% (1917/26 252)	1.9% (1239/63 869)	6.3% (619/9818)
Blood transfusion	1.9% (1682/87 345)	8.8% (2234/25 501)	2.6% (854/32 893)	9.6% (1740/18 217)	12.2% (2172/17 799)	23.2% (6087/26 252)	0.6% (399/63 869)	7.6% (749/9818)
C. difficile positive	0.3% (296/87 345)	1.1% (271/25 501)	1.1% (362/32 893)	2.5% (450/18 217)	1.3% (227/17 799)	1.8% (480/26 252)	0.2% (122/63 869)	0.7% (66/9818)
Non‐contrast chest CT following XRT	1.6% (847/53 272)	3.0% (496/16 658)	2.1% (128/6022)	4.1% (216/5229)	10.3% (977/9523)	11.3% (2339/20 735)	1.1% (422/37 290)	4.9% (218/4425)
Elevated creatinine	0.1% (131/97 462)	0.5% (136/26 791)	0.3% (85/33 712)	0.8% (158/18 671)	0.6% (125/21 142)	1.0% (315/30 169)	0.1% (67/84 140)	0.4% (43/10 800)
Hypoxemia/low oximetry	1.0% (992/97 462)	4.0% (1078/26 791)	2.8% (937/33 712)	5.9% (1103/18 671)	12.2% (2572/21 142)	16.3% (4927/30 169)	1.2% (1050/84 140)	4.6% (493/10 800)
Contact precautions/isolation	6.0% (2630/43 825)	9.5% (2147/22 616)	4.1% (483/11 816)	6.0% (931/15 614)	6.2% (732/11 732)	8.0% (1976/24 672)	1.2% (367/29 511)	3.6% (322/8924)
Nasogastric tube	< 11 events	< 11 events	0.5% (124/27 078)	0.6% (68/10 600)	< 11 events	< 11 events	0.1% (29/38 298)	< 11 events
Nephrology consult	0.3% (254/97 462)	1.3% (350/26 791)	1.4% (485/33 712)	3.1% (577/18 671)	1.3% (279/21 142)	2.1% (645/30 169)	0.6% (541/84 140)	2.6% (284/10 800)
Neutropenic fever	4.0% (1765/43 825)	6.8% (1535/22 616)	4.5% (528/11 816)	5.7% (894/15 614)	6.7% (788/11 732)	7.4% (1814/24 672)	0.6% (173/29 511)	2.6% (231/8924)
Percutaneous drain	0% (25/68 318)	0.1% (18/15 425)	0.8% (206/27 078)	2.4% (252/10 600)	0.6% (48/8144)	0.5% (25/5160)	0.3% (96/38 298)	0.6% (12/2060)
Abnormal serum potassium	2.5% (2218/87 345)	8.7% (2211/25 501)	7.3% (2417/32 893)	17.7% (3233/18 217)	8.1% (1445/17 799)	13.9% (3656/26 252)	1.8% (1135/63 869)	6.9% (674/9818)
Press ulcer	0.3% (230/68 318)	0.6% (85/15 425)	1.2% (313/27 078)	1.5% (154/10 600)	1.0% (78/8144)	0.9% (45/5160)	0.2% (95/38 298)	0.8% (17/2060)
Return to OR or IR	0.1% (63/68 318)	0.2% (37/15 425)	2.4% (638/27 078)	3.7% (392/10 600)	0.4% (33/8144)	0.4% (21/5160)	0.6% (229/38 298)	0.7% (14/2060)

Note

Values shown in the table are prevalence rates and the number of patients with a trigger within an exposure window divided by the number exposed in parentheses.

### 3.3 Multiple triggers

Certain patients experienced a particularly high number of trigger events, although it is important to note that a single adverse event could give rise to multiple triggers. As shown in Table 4, 19% of patients had at least one event. Among patients with nonmetastatic disease, 4% had one trigger event over the course of the year and 8% had two or more. Among those with metastatic disease, 10% had one trigger event and 29% had two or more. Individual patients with lung cancer had a particularly high burden of trigger events; one‐quarter of patients with nonmetastatic disease and one‐third of those with advanced disease experienced multiple triggers.

**Table 4:** Number of triggers per patient within 1 y, by cancer type and metastatic status

Cancer type	N	Nonmetastatic n = 236 456			Metastatic n = 86 431			All n = 322 887		
		No trigger	1 trigger	>1 triggers	No trigger	1 trigger	>1 triggers	No trigger	1 trigger	>1 triggers
Breast	124 253	89.8%	6.7%	3.5%	69.1%	16.6%	14.3%	85.3%	8.8%	5.9%
Colorectal	52 383	80.8%	12.1%	7.1%	58.6%	20.3%	21.1%	72.8%	15.0%	12.1%
Lung	51 311	67.2%	18.4%	14.4%	49.8%	23.8%	26.4%	57.0%	21.6%	21.4%
Prostate	94 940	94.1%	4.3%	1.7%	75.4%	13.4%	11.1%	92.0%	5.3%	2.7%
Total	322 887	88.0%	7.6%	4.4%	60.9%	19.5%	19.6%	80.7%	10.8%	8.4%

Note

Percentages may not add to 100% due to rounding.

### 3.4 Risk factors associated with triggers

Table 5 displays trigger prevalence by subject characteristics, stratified by cancer type. Triggers were less prevalent among young patients, men, whites, families with incomes over $150 000 per year, and patients with some college education. These differences were small but statistically significant (*P* < .001), perhaps reflecting certain patients’ better access to care, earlier cancer detection, and lower intensity therapy.

**Table 5:** Cohort characteristics by trigger prevalence

Characteristic	No trigger	At least one trigger	Overall
N	260 700	62 187	322 887
Age [mean(SD)]	63.3 (11.6)	64.1 (11.8)	63.5 (11.6)
Sex [n (%)]			
Male	123 171 (47.3%)	26 555 (42.7%)	149 726 (46.4%)
Female	137 529 (52.7%)	35 632 (57.3%)	173 161 (53.6%)
Race/Ethnicity [n (%)]			
Missing/Unknown	86 541 (33.2%)	21 791 (35.0%)	108 332 (33.6%)
Asian	4103 (1.6%)	810 (1.3%)	4913 (1.5%)
Black	19 738 (7.6%)	5110 (8.2%)	24 848 (7.7%)
Hispanic	10 394 (4.0%)	2191 (3.5%)	12 585 (3.9%)
White	139 924 (53.7%)	32 285 (51.9%)	172 209 (53.3%)
Annual household income [n (%)]			
Unknown	101 758 (39.0%)	26 741 (43.0%)	128 499 (39.8%)
<$25K	32 266 (12.4%)	9652 (15.5%)	41 918 (13.0%)
$24K‐$149K	42 022 (16.1%)	10 394 (16.7%)	52 416 (16.2%)
$150K‐249K	46 225 (17.7%)	9064 (14.6%)	55 289 (17.1%)
$250K‐$499K	23 263 (8.9%)	4104 (6.6%)	27 367 (8.5%)
$500K+	15 166 (5.8%)	2232 (3.6%)	17 398 (5.4%)
Education [n (%)]			
Missing/Unknown	80 855 (31.0%)	20 539 (33.0%)	101 394 (31.4%)
Less than 12th grade	456 (0.2%)	140 (0.2%)	596 (0.2%)
High school diploma	45 256 (17.4%)	12 839 (20.6%)	58 095 (18.0%)
Less than bachelor degree	97 481 (37.4%)	22 228 (35.7%)	119 709 (37.1%)
Bachelor degree plus	36 652 (14.1%)	6441 (10.4%)	43 093 (13.3%)
Insurance [n (%)]			
Private insurance [n (%)]	202 110 (77.5%)	46 960 (75.5%)	249 070 (77.1%)
Medicare Advantage [n (%)]	58 590 (22.5%)	15 227 (24.5%)	73 817 (22.9%)
Clinical characteristics			
Metastatic disease [n (%)]	52 607 (20.2%)	33 824 (54.4%)	86 431 (26.8%)
Charlson index^a^ [mean (SD)]	2.5 (1.7)	3.0 (1.9)	2.6 (1.7)
Rehospitalized w/in 1 y [n (%)]	97 642 (37.5%)	42 075 (67.7%)	139 717 (43.3%)
Hospital days [mean (SD)]	5.4 (8.6)	11.7 (15.2)	7.3 (11.4)
Treatment type [n (%)]			
Chemotherapy only	42 069 (16.1%)	15 343 (24.7%)	57 412 (17.8%)
Radiation therapy only	38 853 (14.9%)	2340 (3.8%)	41 193 (12.8%)
Surgery only	78 754 (30.2%)	5384 (8.7%)	84 138 (26.1%)
Multimodality	101 024 (38.8%)	39 120 (62.9%)	140 144 (43.4%)

Note

Percentages may not add to 100% due to rounding.

a NIH measure for cancer patients’ modification. https://healthcaredelivery.cancer.gov/seermedicare/considerations/calculation.html

## 4 DISCUSSION

In this retrospective cohort study of 322 887 patients with breast, lung, colorectal, and prostate cancer treated for an initial course of cancer‐directed therapy, we found that one in five patients had a “trigger” event that indicated a likely treatment‐related AE. The most common triggers included laboratory abnormalities of bicarbonate and potassium, need for blood transfusion, hypoxemia, neutropenic fever, and contact precautions. The burden of event triggers fell disproportionately on patients with lung and colorectal cancer compared to those with breast or prostate cancer, and among those with metastatic disease. The prevalence of trigger events among patients with metastatic disease was more than triple the rate among those with nonmetastatic disease (39.1% vs 12.0%), and as high as 50.2% in patients with metastatic lung cancer. Nearly three in four patients with a trigger had two or more such events.

Triggers are clinical indicators that signal the possibility of treatment‐related injury, and therefore, the trigger rate needs to be adjusted by the probability that the trigger denoted an actual harm event. The general medicine literature describes PPVs of 17%‐45% based on physician chart review as the gold standard.^9^, ^10^, ^27^, ^28^, ^29^, ^30^ Since the number of trigger events may overestimate the number of actual AEs, we estimated the incidence of AEs and preventable AEs present in the cohort using PPVs calculated in the original MSK medical record review‐based developmental study. Using the MSK trigger‐specific PPVs, the present study found an estimated 97 521 AEs and 24 915 preventable AEs affecting 30.2% and 7.7% of cancer patients in this cohort, respectively.

Direct comparison of our findings with research on AEs in cancer care is challenging, given the use of inconsistent and disparate research methods. Comparison with toxicity rates in cancer clinical trials is also problematic, as trials generally exclude low‐severity “expected” toxicities. The most rigorously conducted studies reported single‐institution medication error rates of 4%‐7% among adult cancer patients.^2^, ^3^ Only 1%‐2% had the potential for harm, and the majority of errors among patients undergoing cancer treatment were due to non‐chemotherapy medications.^5^ Of those potentially harmful errors, still fewer reached the patient and resulted in a preventable injury. If our estimates are accurate, then previous studies of cancer treatment‐related errors may have underestimated the rate of preventable treatment‐related AEs by at least an order of magnitude.

Multiple investigators have documented a discrepancy between the number of AEs identified using trigger tools compared to alternative methods, including the use of traditional clinician‐reporting tools. Trigger‐assisted AE detection identifies dramatically more events than those detected using other approaches,^31^, ^32^, ^33^ and automated trigger tools that are embedded in the electronic medical record may yield event rates as high as 40%.^34^, ^35^, ^36^

In studying the prevalence of cancer‐specific triggers, we sought to examine the association between triggers and patients’ sociodemographic characteristics. We reasoned that triggers might be more common among patients from racial or ethnic minorities and among those with lower socioeconomic status and educational attainment because of limited access or obstacles to care. The data supported this hypothesis, although the between‐group differences were small. While research links poor outcomes with delayed cancer diagnosis, patient safety researchers have not demonstrated a compelling link between adverse events and race or ethnicity. However, the current evidence base is sparse and inconclusive.^37^

This study's strengths include its large sample size, diverse patient population, and longitudinal cohort spanning inpatient and ambulatory care. It is also subject to several limitations. OLDW includes information about commercial and Medicare Advantage patients and our findings, therefore, may not be generalizable to a Medicare Fee for Service or Medicaid cohort. The use of claims data has inherent limitations. Certain diagnostic and treatment codes lack specificity. Claims‐based algorithms may fail to distinguish accurately patients with late recurrences or to characterize those with metastatic disease, problems we sought to minimize by drawing on well‐validated coding algorithms. Given the burden of disease‐related morbidity in cancer care, there is expected confounding of AEs related to either disease or treatment. We attempted to address this inherent challenge by linking treatment exposure to trigger events by type of exposure, timing of event relative to exposure, and duration of event. While this approach improved the likelihood that a given treatment caused a trigger event, perfect attribution of trigger to treatment would require expert chart review—a project that we hope to undertake in the future. Finally, it is important to note that triggers, though they flag a broad spectrum of events, are neither comprehensive nor exhaustive. Narrowly constructed tools that link specific treatment regimens for stage‐specific cancer types may be better at detecting certain types of events, such as chemotherapy‐related AEs.^38^ Triggers detect a subset of all potential harms rather than the universe of AEs.^39^

In conclusion, a claims‐based oncology‐specific trigger tool appears both feasible to construct and instructive in its results. Treatment‐related triggers are common in cancer care, suggesting a significant burden of anticipated and potentially unexpected and even preventable AEs. The trigger burden falls unevenly across patients by disease, metastatic status, treatment type, and socioeconomic status, affecting exactly those patients most vulnerable to harm. Additional research is needed to assess the association of cancer triggers with key clinical outcomes such as disease‐attributable and overall mortality, resource utilization, and patient‐centered outcomes. Oncology‐specific triggers offer the opportunity to better understand and characterize the nature and extent of AEs in cancer care, and to inform interventions that may reduce the burden of harm among patients with cancer.

## Data Availability

The data that support the findings of this study are available from OptumLabs®. Restrictions apply to the availability of these data, which were used under license for this study.

## APPENDIX 1 Oncology triggers, by exposure and timing window

**Table:**

Trigger	Exposure	Timing (days)	Rationale	Window (days)	Notes
Anticoagulation	Surgery, chemotherapy	30	Treatment‐related venous thromboembolism due to immobility, thrombophilia	180	Prolonged therapy (3‐6 mo) for venous thromboembolism
Bacteremia/ positive blood culture	Surgery, chemotherapy	30	Post‐op or neutropenia‐related infection	30	Normally 14‐d course, but this allows for complicated or device‐related infections
Abnormal serum bicarbonate	surgery, chemotherapy	14	Perioperative fluid management, chemotherapy‐related toxicity	14	Same as abnormal serum potassium
Blood transfusion (1)	Surgery	7	Perioperative bleeding	7	Short window post‐op
Blood transfusion (2)	Chemotherapy	30	Chemotherapy‐related bone marrow toxicity	30	Longer time window
C. difficile positive	Surgery, chemotherapy	30	Post‐antibiotic exposure	30	Toxin takes weeks to resolve, at least
Non‐contrast chest CT following XRT	XRT	30	XRT‐related inflammation	30	
Elevated serum creatinine	All	30	See Nephrology consult	30	See Nephrology consult
Hypoxemia/low oximetry (1)	Surgery	7	Fluid shifts	7	Post‐op splinting or fluid shifts
Hypoxemia/low oximetry (2)	XRT	30	Radiation pneumonitis	60	
Hypoxemia/low oximetry (3)	Chemotherapy	90	Chemotherapy‐related toxicity	90	Bleomycin toxicity can present late
Contact precautions/ isolation	Chemotherapy	30	Presumes infection	30	See Neutropenic fever
Nasogastric tube not placed in OR	Surgery	7	Postoperative ileus	7	In chemotherapy patients, tube more likely related to disease progression
Nephrology consult	All	30	Chemotherapy‐related renal toxicity or surgery/XRT‐related dehydration and azotemia, antibiotic toxicity	30	Could argue for shorter window, but debilitation and kidney injury may take time to resolve
Neutropenic fever	Chemotherapy	30	Chemotherapy‐related neutropenia	30	Normally 14‐dcourse, but this allows for complicated or device‐related infections
Percutaneous drain	Surgery, IR	30	Post‐procedural infection	30	May require extended treatment
Abnormal serum potassium	Surgery, chemotherapy	14	See Abnormal serum bicarbonate	14	Shorter time window for surgery and longer for chemotherapy, but fluctuating values during a course of treatment warrant a 2‐wk window
Pressure ulcer	Surgery	30	Post‐op pressure ulcer	30	
Return to OR or IR	Surgery, interventional radiology	30	Post‐procedural complication	30	Assumes that multiple returns to operating room may be needed to address staged procedure or infection

Abbreviation: CT, computed tomography; IR, interventional radiology; OR, operating room; XRT, radiation therapy.
